# Dynamic Electrochemical Impedance Spectroscopy in Galvanostatic Mode as a Tool for Passive Layer State Monitoring in a Chloride Solution Under a Mechanical Load

**DOI:** 10.3390/ma18010167

**Published:** 2025-01-03

**Authors:** Mateusz Cieślik, Juliusz Orlikowski, Stefan Krakowiak, Krzysztof Żakowski

**Affiliations:** 1 Institute of Nanotechnology and Materials Engineering, Faculty of Applied Science and Mathematics, Gdansk University of Technology, Narutowicza 11/12 Str., 80-233 Gdansk, Poland; mateusz.cieslik@pg.edu.pl; 2 Department of Corrosion and Electrochemistry, Faculty of Chemistry, Gdansk University of Technology, Narutowicza 11/12 Str., 80-233 Gdansk, Poland; juliusz.orlikowski@pg.edu.pl (J.O.); krzysztof.zakowski@pg.edu.pl (K.Ż.)

**Keywords:** passive layer, electrochemistry, g-DEIS, stainless steel

## Abstract

Mechanical stress is one of the factors influencing the initiation of pitting corrosion and deterioration of the protective properties of the passive layer on stainless steel. The tests carried out on AISI 304L stainless steel showed that, in the 3.5% NaCl environment for samples loaded in the elastic and plastic range, no pitting corrosion initiation was observed. Only mechanical damage of the passive layer occurred. Galvanodynamic electrochemical impedance spectroscopy (g-DEIS) was used as the measuring technique. This technique ensures the monitoring of corrosion processes at zero external current (I_DC_ = 0) and no potential perturbation of the system. It also allows one to perform many measurements, so that short-term changes such as cracking of the layer and its repassivation are possible to monitor.

## 1. Introduction

The mechanism of pitting corrosion is difficult to study because of its local nature. The existing models of pitting corrosion can be divided into two groups: one assumes that pitting initiation occurs at the metal/oxide layer interface, while the other assumes that the pitting initiation site is located at the oxide/electrolyte interface. In both cases, the properties of the passive layer play a decisive role [1]. Anions (most often chloride) accelerate the process of destruction of the passive layer on metals in aqueous solutions, but the exact mechanism of their action is not known. Some authors indicate that the aggressiveness of these ions results from their small size, high diffusion coefficient, and the formation of hydrolyzing salts with alloy components, creating a strongly acidic environment [2,3,4,5]. The result of pitting corrosion development is sudden and severe structural damage [3]. The presence of chloride ions in a corrosive environment causes a passive layer discontinuity on the metal surface. Easily soluble metal–chlorine complexes are formed [3], which causes the local lowering of the pH inside the pit [4]. Pit growth is controlled by the diffusion of metal ions from the pit into the bulk solution. The pit growth rate increases with the diffusion rate [5]. There are three stages of pitting corrosion attack: initiation, metastable propagation, and stable propagation [6,7].

The kinetics of pit growth depend on the metal composition, ohmic potential drop inside the pit, electrolyte composition inside the pit, the porosity, strength, and elasticity of the passive layer, the electrochemical potential at the pit bottom, and the pH difference inside and outside the pit [8]. The main factors affecting the formation of pits are associated with the environmental conditions and structure of the material. Halides effectively attack passive layers, leading to pit initiation and intensive local dissolution of the underlying metal.

Several mechanisms have been proposed to explain the effect of chlorides on passive layer breakdown and pit initiation [7]. Three main mechanisms are discussed in the literature: the penetration mechanism [9], the film-breaking mechanism [10,11], and the adsorption mechanism [12]. Strong depolarizers, such as Fe^3+^, Cu^2+^, and Hg^2+^, may also initiate pit formation [13]. Pitting formation is also affected by the critical pitting temperature (CPT), which is described in detail by Frankel, G.S. [14]. Higher temperatures favour pit stabilization and make repassivation difficult [15].

Another factor facilitating the initiation of pitting corrosion is the state of the metal surface preparation. The probability of pitting corrosion expressed as the value of the pitting nucleation potential (Enp) varies linearly with the increase in the grit size of the sandpaper used for surface preparation, which is consistent with the assumption that the pitting corrosion mechanism is a diffusion-controlled process [8,16].

Material factors affecting pitting corrosion are related to the alloy composition and microstructure. In the vast majority of cases, pitting initiates in some physicochemical heterogeneity on the surface, such as inclusions, grain boundaries, defects, mechanical damage, local stress gradient, or dislocations [7,15]. Sulphide inclusions in the form of MnS play a major role in the initiation of pits in stainless steel [17,18]. The chemical composition of an alloy is crucial in terms of the structure and properties of the passive layer. The condition and quality of the passive layer formed on stainless steels are closely related to the chromium content in the alloy [6]. The potential for corrosion with an increasing chromium content increases significantly, especially in the case of steels in which the content of this element is higher than the minimum necessary to create a passive state. The potential for corrosion with an increasing chromium content increases significantly, especially in the case of steels in which the content of this element is higher than the minimum necessary to create a passive state. The addition of chromium to steel significantly changes the nature of the oxide formed on the steel surface. With an increasing chromium content, the oxide becomes more amorphous and therefore homogeneous, with fewer defects [19].

The content of this element significantly influences, alongside molybdenum and nitrogen, the value of the PREN coefficient (pitting resistance equivalent number).

Mechanical stress acting on stainless steel structures operating in real conditions causes the discontinuity of the passive layer, which leads to the initiation of pitting corrosion at the sites of local heterogeneity [20], and has an effect on corrosion fatigue or stress corrosion cracking [21,22]. Yang et al. [23] found that tensile stresses applied to type 304 stainless steel in chlorides in the presence of hydrogen reduce the critical chloride concentration for the breakdown of a passive film. Some research focuses on the enhancement of passive layer reactivity in the elastic domain under static mechanical stress [24]. Sidane et al. [25] studied the behaviour of 304L stainless steel and found that the kinetic constant for the oxidation of K_3_Fe(CN)_6_ in 0.5 M K_2_SO_4_ on the substrate increased significantly with increasing stress magnitudes. Vignal et al. [26] carried out research on the semiconducting behaviour of 316L stainless steel under elastic stress in a solution of pH 3 containing 0.02M NaCl and discovered that high elastic stress caused the passive films to be heavily doped, suggesting that passive film conductivity was increased. Also, heavily doped films were sensitive to pitting corrosion. Wu and Singh [27] reported that elastic tensile stresses hinder the pitting resistance of 304 stainless steel, because stresses in the pit’s proximity probably accelerate salt-film repair, hence limiting repassivation and stabilizing pit growth. Feng et al. [28] examined the passive behaviour of deformed 304 stainless steel samples in a saturated calcium hydroxide solution. The passive films on seriously deformed stainless steel became heavily doped. Simultaneously, the Fe^2+^ ion content in the films decreased, whereas the Fe^3+^ ion content increased with an increasing degree of deformation but did not have a significant influence on the content of Cr cations.

According to reports that can be found in the literature, mechanical stress accelerates the pitting corrosion of the passive layer of austenitic steels [22,29,30]. Research conducted by the authors of this manuscript focused on a non-invasive measurement technique for monitoring the cracking of the passive layer under the influence of stress in the elastic and plastic range of the tested 304L stainless steel. Since the use of classical electrochemical impedance spectroscopy (EIS) does not provide reliable results in the study of dynamic processes due to the long time required to obtain a single spectrum, the galvanostatic dynamic impedance spectroscopy (g-DEIS) method was used. Galvanostatic dynamic impedance spectroscopy is a development of classic electrochemical impedance spectroscopy (EIS) where the basic difference is the dynamic nature of the test. A detailed description of the research methodology used in the submitted publication is included in [31,32,33,34]. The constant current in this measuring method is kept at zero, which does not interfere with the electrochemical properties of the tested steel. The purpose of the work was to check the resistance of the passive layer to mechanical damage, determine the moment of layer breakage, and check whether it was possible to repassivate the layer despite the constant stress. Additionally, a tool to monitor the condition of the passive layer under mechanical stress was developed.

## 2. Materials and Methods

### 2.1. Sample Preparation

**Figure 1:**
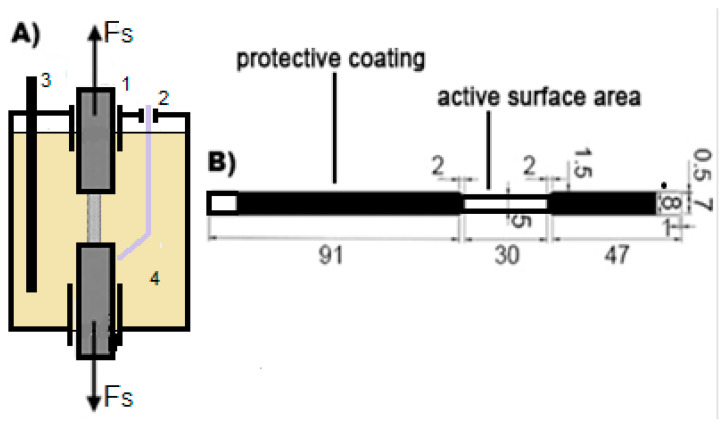
Measurement of the electrochemical cell (**A**): 1—tested sample; 2—reference electrode, Ag/AgCl; 3—counter electrode; and 4—solution, 3.5% NaCl. Dimensions of the tested sample (**B**).

The tested samples were made of AISI 304L stainless steel (304L SS) in the form of bars. The dimensions of the sample are shown in Figure 1B. Unrolled parts of the cylinders were sandblasted for better surface development and coated with chlorinated rubber coating enamel to prevent them from corrosion and reduce the active area of the sample. The exposed area of the samples was prepared by grinding with 500–2000-grade abrasive papers and polished using diamond suspensions. The polished surfaces were cleaned using acetone and triple-distilled water and dried quickly in a stream of warm air. The samples were stored in a desiccator until the measurements were started. The chemical composition of the steel was confirmed using an emission spectrometer (OES), Spectrolab LAB05 (Spectrolab, Sylmar, CA, USA). The results are presented in Table 1.

Based on the chemical composition of the investigated stainless steel, the PREN value was also calculated using the following formula:

PREN = % Cr + 3.3% Mo + 30% N

where % Cr, % Mo, and % N indicate the content of individual alloying components expressed in % by weight.

### 2.2. Tensile Stress Test

Samples immersed in 3.5% NaCl were exposed to a constant load during the electrochemical measurements. A diagram of the measurement electrochemical cell is shown in Figure 1A.

**Figure 2:**
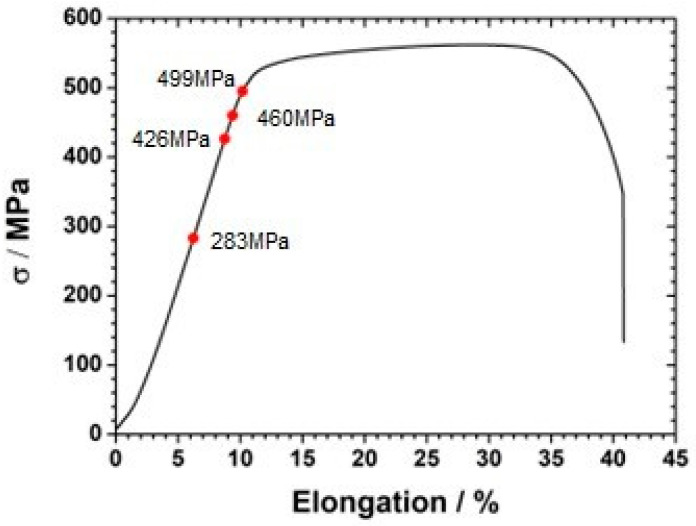
Tensile strength as a function of elongation for samples made of 304L stainless steel. The marked points show the maximum stresses for which the electrochemical measurement was carried out.

The sample loading forces were 283 MPa (in terms of the limit of elasticity), 426 MPa, 460 MPa, and 495 MPa (above the yield point), respectively, visible in Figure 2. The tests were carried out on a tensile testing machine Roell Z030 (Zwick, Ulm, Germany). The average yield point was 398 MPa, and the average strength limit value was 559 MPa.

### 2.3. Electrochemical Measurements

Electrochemical measurements were carried out in a three-electrode system. The working electrode was 304L stainless steel (E_w_), with Ag/AgCl as a reference electrode (E_r_) and platinum mesh as a counter electrode (E_c_). The exposed area of the 304L SS sample was 4.7 cm^2^.

Cyclic polarization measurements were performed in 3.5% NaCl in natural oxidation conditions of a solution. The test solution was prepared by dissolving weighed amounts of analytical-grade sodium chloride in triple-distilled water. Electrochemical studies were performed using a Reference 600 potentiostat/galvanostat (Gamry Instruments, Warminster, PA, USA), after sample preconditioning for 30 min to determine the corrosion potential of 304L SS. The polarization scans were recorded around −0.1 V under E_CORR_ (Table 2) to the potential, at which the current density was equal to 1 mA/cm^2^. After reaching this current density value, the polarization was carried out in a cathodic direction. The scan rate was 1 mV/s. The pitting potentials were obtained by observing the anodic polarization curve along which the anodic current increased to 10 µA × cm^−2^.

GDEIS (galvanostatic dynamic electrochemical impedance spectroscopy) is a variation of DEIS (dynamic electrochemical impedance spectroscopy). Both techniques involve stimulating the tested system with an excitation signal composed of sinusoidal signals of specific frequencies and a possible constant component. The difference between them is that, in DEIS, the excitation is in the potential form, and the response is in the current form, while, in GDEIS, the excitation is a current signal, and the response is a potential signal resulting from the characteristics of the tested system. By analyzing the excitation and response signals using the Short-Time Fourier Transform (STFT), it is possible to determine the impedance of the tested system at each frequency used for excitation in one period time (analysis window). To simplify this, in one time window/period time, which in this test lasted 1 s, one could obtain one full impedance spectrum. The measurement is continuous, and the result is a set of spectra as a function of time, which, in this study, were also a function of mechanical stress.

The stimulating packet consisted of 23 frequencies, which were prime numbers. This avoided harmonic errors in the response signal. The minimum frequency used affected the length of the analysis window. In the case of a 1 s window, the lowest frequency was f = 3 Hz. The highest frequency in the excitation signal was f = 45,007 Hz. The frequency coverage was four orders of magnitude.

Measurements were performed without the DC component, thus avoiding the introduction of shock electrochemical stimulation. The tested system had its equilibrium potential all the time, because the galvanostat kept the current at DC = 0 A.

The measurement set consisted of a Gamry REF600 potentiostat/galvanostat (Gamry Instruments, USA), coupled with National Instruments DAQ PXI (NI, Austin, Texas, USA) cards used to generate the excitation signal, acquire the response signal, and analyze the data. Everything was controlled by custom software (Dr Rust, Gdansk, Poland) written in the NI LabView (NI, Austin, Texas, USA) environment. To obtain the most accurate spectra, the signals were sampled with an ADC converter (NI, Austin, Texas, USA)with a resolution of 24 bits at a frequency of 800 kS/s. Gamry equipment was used because its measuring system had a floating ground, thanks to which it was possible to eliminate the interference from the tensile test machine. All measurements were carried out at room temperature (about 25 °C).

## 3. Results

**Figure 3:**
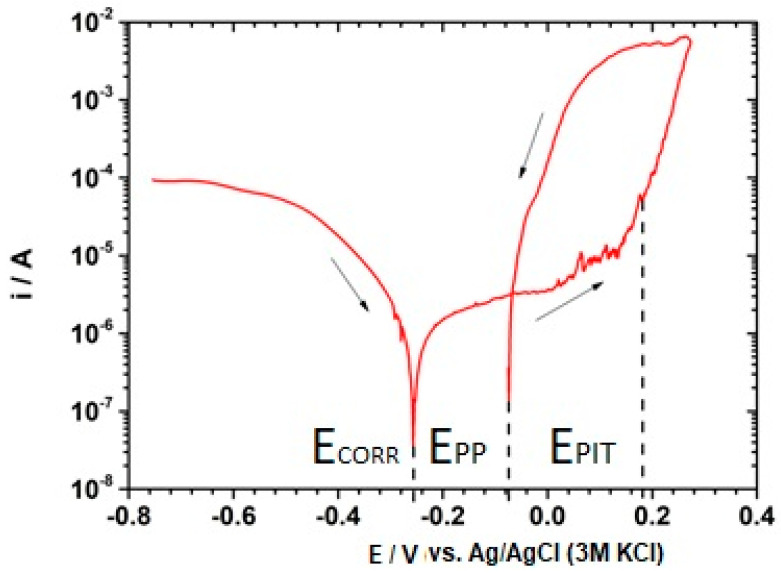
Cyclic polarization curve 304L-type stainless steel exposed to 3.5% NaCl.

Figure 3 shows an example of a cyclic polarization curve for AISI 304L-type stainless steel in 3.5% NaCl solution. The arrows indicate the direction of potential changes.

The exemplary curve obtained for cyclic polarization, shown in Figure 3, indicates that AISI 304L stainless steel is susceptible to pitting corrosion in an environment of 3.5% NaCl. This is indicated by the hysteresis loop appearing on the polarization curve. In the range from the corrosion potential to a potential of approximately 0.06 V, stainless steel is characterized by a stable passive state. When this potential value is exceeded, current fluctuations related to the formation and repassivation of unstable pits are visible. Considering the statistical nature of pitting corrosion [30], five cyclic polarization curves were obtained. Based on the cyclic polarization curves, the characteristic potentials of pitting corrosion were determined. The results of the analysis of potential parameters from a series of cyclic polarization measurements are presented in Table 2 in the form of the average potential value and standard deviation. The corrosion potential is characterized by a relatively low value for stainless steels, and the value of the critical pitting corrosion potential at an average level of 0.18 V indicates a significant susceptibility of type 304 steel to pitting corrosion in a 3.5% NaCl environment.

**Figure 4:**
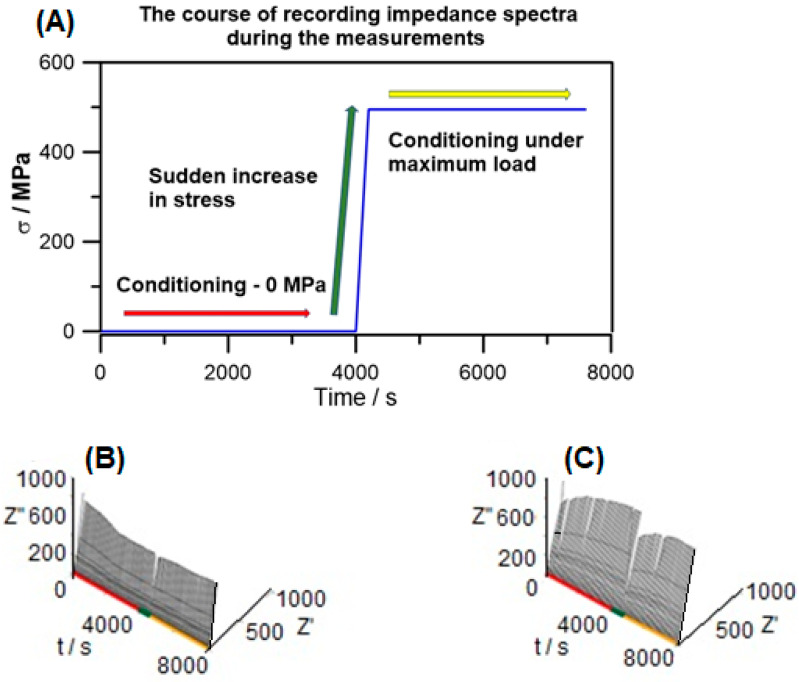
The course of the experiment (**A**) and impedance spectra in the Nyquist plot of g-DEIS results registered before mechanical process, during the mechanical process, and after the mechanical process for which the maximum stress was (**B**) 283MPa and (**C**) 426MPa.

From the values of individual potentials in Table 2, it can be concluded that 304L steel is repassivated in a solution of 3.5% NaCl. The return curve cuts the passive state at a potential of about −0.07 V, determining the value of the protective potential. This indicates that the pitting corrosion process may not occur spontaneously in this environment.

g-DEIS measurement results are presented in the form of impedance spectrograms as a function of measurement time, and the graphs are shown in Figure 4.

During a sudden increase in stress, a decrease in impedance is observed. The passive layer is destroyed under the influence of the applied stress. After reaching the maximum load and conditioning under the maximum load, the impedance value begins to increase. This proves that the steel surface is repassivated in accordance with the theory of a self-healing oxide layer on stainless steels. The state of the steel surface undergoes a transition from a passive state to an active state. For the analysis of impedance spectra, an electric equivalent circuit was used. Changes in the surface condition were considered.

**Figure 5:**
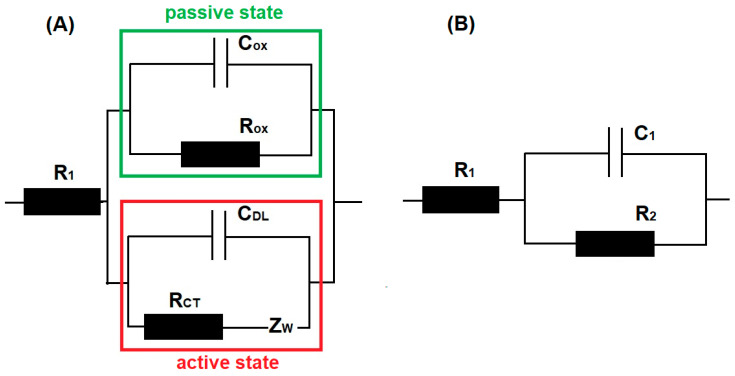
Electric equivalent circuit (**A**) before simplification—R_1_, electrolyte resistance; R_OX_, passive layer resistance; C_OX_, passive layer capacitance; R_CT_, charge transfer resistance; C_DL_, double-layer capacitance; and Z_W_, Warburg impedance—and (**B**) after simplification, with R_1_, electrolyte resistance; R_2_, charge transfer resistance; C_1_, capacitance.

In the selection of the electrical equivalent circuit, it was assumed that the surface of stainless steel had areas covered with a passive layer and areas where corrosion occurred. The R_ox_ and C_ox_ values shown in Figure 5A describe the resistance and capacity of the passive layer, respectively. Corrosion processes occur on surfaces without a passive layer, described by the charge transfer resistance, double-layer capacity, and Warburg impedance. The precise separation and differentiation of current flow in these two paths are impossible, so for the analysis of impedance spectra, a simplified electric equivalent circuit was utilized (Figure 5B). The selection of the proper equivalent circuit fitting to spectra in the time of the measurement was necessary for the automation of analysis for a monitoring system. The first simplification for capacitance measurement is the sum of passive layer capacitance and double-layer capacitance [34]. The value of resistance R_2_ presented in Figure 5B is the resultant value of resistances R_OX_ and R_CT_.

**Figure 6:**
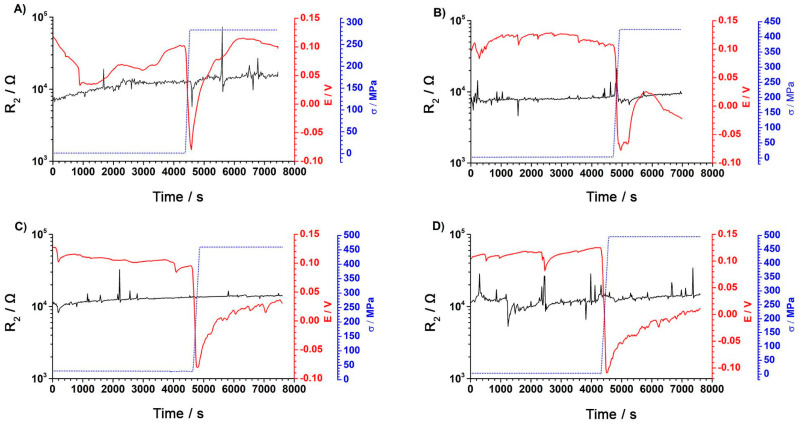
R_2_ resistance changes as a function of time for a final stress of (**A**) 283MPa, (**B**) 426MPa, (**C**) 460MPa, and (**D**) 495MPa. Resistance R_2_ value: black line; potential value: red line; and tensile stress process: blue dotted line.

The figures below show the dependence of changes in the elements of the electrical equivalent circuit as a function of the measurement time. Figure 6 presents the changes in R_2_ resistance.

**Figure 7:**
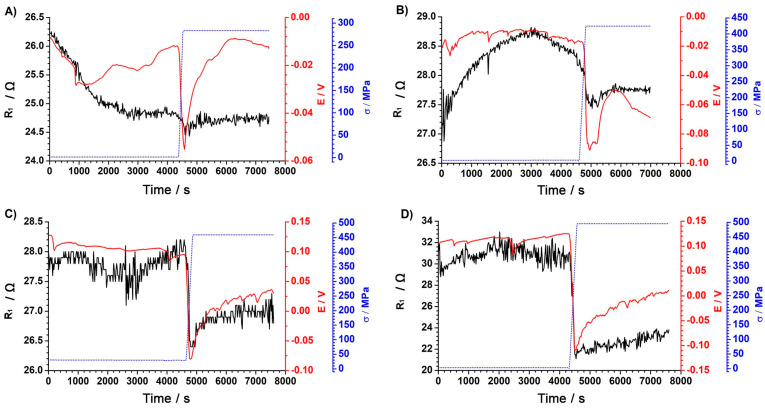
R_1_ resistance changes as a function of time for a final stress of (**A**) 283MPa, (**B**) 426MPa, (**C**) 460MPa, and (**D**) 495MPa. Resistance R_1_ value: black line; potential value: red line; and tensile stress process: blue dotted line.

The results presented in Figure 6A–D indicate that the resistance values R_2_ do not change significantly during initial exposure without a load or during stretching and measurement at a constant load. The results indicate that the corrosion process did not occur, because the R_CT_ charge transfer resistance was much smaller than the resistance of the R_OX_ passive layer [35]. The potential values decreased as the tensile stress increased, which may indicate a deterioration of the barrier in comparison to the value of resistance R_2_ of an equivalent circuit. Figure 7 presents the changes in R_1_ electrolyte resistance.

**Figure 8:**
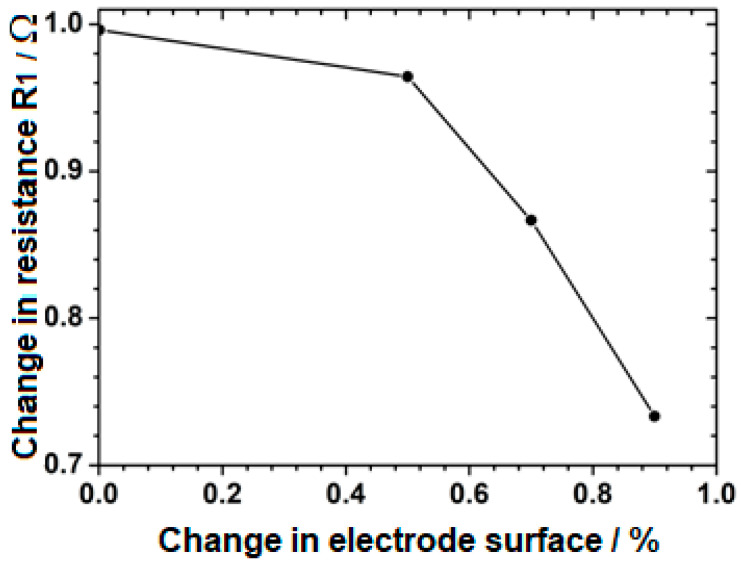
Correlation between changes in resistance R_1_ and electrode surface for each applied stress. The surface area was measured using a calliper.

Analyzing the trend of changes in resistance R_1_, a characteristic value decrease in a stepwise manner when the stress appeared was visible. This was particularly evident in the figures, which showed the changes for 460 MPa and 495 MPa tensile stress values. Such a behaviour may have resulted from the loss of continuity of the passive layer and a change in the sample surface being tested. During the implementation of mechanical tests, the electrode surface area increased in the case of tests carried out in the plastic range (426 MPa, 460 MPa, and 495 MPa). The changes in resistance R_1_, correlated with the changes in the surface of the tested electrodes with increasing stress, are shown in Figure 8.

**Figure 9:**
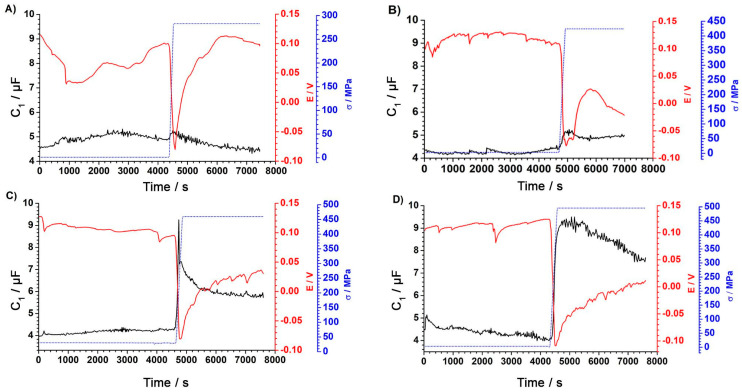
C_1_ capacitance changes as a function of time for a final stress of (**A**) 283MPa, (**B**) 426MPa, (**C**) 460MPa, and (**D**) 495MPa. Capacitance C_1_ value—black line; potential value—red line; and tensile stress process—blue dotted line.

The analysis showed that there was a clear correlation between the change in the surface of the tested electrode, associated with the mechanical stretching of the sample, and changes in resistance R_1_. This indicates that resistance R_1_ is mainly associated with the electrolyte resistance and its changes. The second factor that influences the R_1_ value is the geometrical changes in the tested sample area. It is not possible to separate the two components of resistance R_1_ into the part responsible for changes in the electrolyte properties and sample geometry, respectively. The capacitance of the passive layer is inversely proportional to its thickness [36]; therefore, as the layer thickness increases, the measured capacity decreases [37,38]. After exceeding the stress of about 254 MPa, a significant increase in the value of C_1_ capacity can be seen. This change indicates a loss of layer continuity because the dielectric permittivity of water is several times higher than the dielectric permittivity of the passive layer [39,40]. Water absorption by the layer causes an increase in dielectric permeability, which, in turn, leads to an increase in layer capacitance [39]. An increase in layer capacity is observed depending on the value of applied stress, as stress increases, the capacity of the passive layer increases. The graph of Figure 9 shows the compatibility of changes in layer capacitance with the changes in potential value. After reaching the maximum stress, the capacitance value begins to decrease. This indicates the process of repassivation of a mechanically damaged passive layer.

Changes in the capacity parameter can be correlated with the lack of changes in resistance R_2_ (Figure 6) and confirm that the pitting corrosion process does not occur, and the layer is mechanically damaged and undergoes self-regeneration. Capacity, C_1_, can be treated as an indicator of the state of the durability of the passive layer on stainless steel.

**Figure 10:**
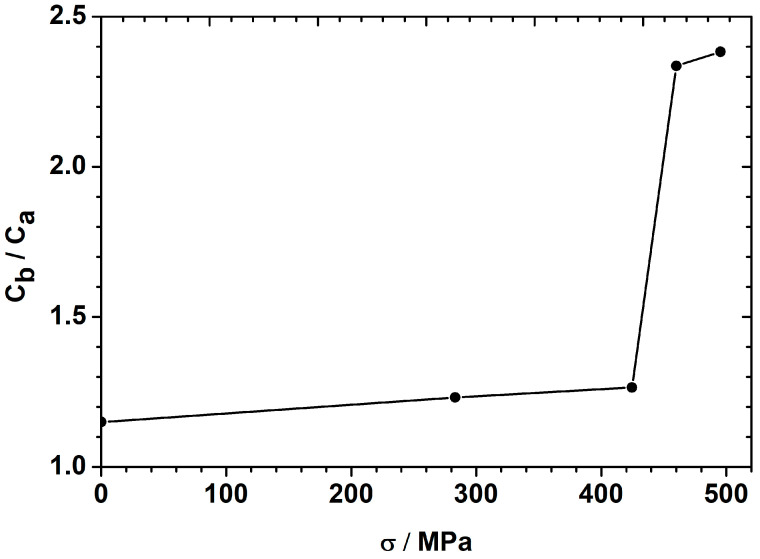
The capacity ratio before and after breaking the passive layer as a function of tensile stress.

This research has shown that capacitance C_1_ can be a parameter for assessing changes in passive layer properties dependent on tensile stress. The parameter was standardized and is shown in Figure 10. To evaluate capacitance changes during passive layer rapture, the capacitance value of the passive layer after the stress process (C_a_) was divided by the capacitance value directly before the stress process (C_b_). Results of the capacity ratio in Figure 10 are shown for stresses for which the stretching process was halted and indicate a clear relationship between changes in capacity as a function of tensile stress. As the stress value increases, there is an increase in the capacity change ratios. There is a sudden change between 426MPa and 460 MPa. For lower stress values and the non-tensile sample, the C_a_/C_b_ ratio is close to one.

## 4. Conclusions

The measurements were carried out under the specified conditions without affecting the electrochemical properties of stainless steel, without sample polarization. It was found that cracking of the passive layer occurred at stresses falling within the range of plastic deformation while its rapid repassivation occurred. The change in capacity was the most selective parameter, indicating degradation of the passive layer. As the stress increased, the level of degradation of the passive layer increased, which was visible in the form of increasing capacity changes. No pitting corrosion was initiated for all applied stresses. The proposed technique allows for the analysis of steel susceptibility to pitting corrosion under mechanical loads.

## Figures and Tables

**Table 1:** Chemical composition (in wt%) of the 304L SS examined samples.

C	Mn	Si	P	S	Cr	Ni	Fe	PREN
0.03	1.70	0.82	0.07	0.04	18.28	8.82	70.21	18.28

**Table 2:** Results of the analysis of cyclic polarization measurements of 304L stainless steel in 3.5% NaCl.

	E/V	Rel. Standard Dev.
Corrosion potential, E_CORR_	−0.26	0.078
Critical pitting potential, E_PIT_	0.18	0.085
Protection potential, E_PP_	−0.07	0.090

## Data Availability

The original contributions presented in the study are included in this article; further inquiries can be directed to the corresponding authors.
